# Efficacy and safety of biosimilar trastuzumab (HLX02) in patients with HER2-positive advanced breast cancer: a retrospective real-world analysis

**DOI:** 10.3389/fonc.2025.1622854

**Published:** 2025-08-01

**Authors:** Xuan Ye, Linlin Wang, Wensheng Liu, Mengmeng Wang, Zihan Guo, Han Shan, Qing Zhai, Qiong Du

**Affiliations:** ^1^ Department of Pharmacy, Fudan University Shanghai Cancer Center, Shanghai, China; ^2^ Department of Oncology, Shanghai Medical College of Fudan University, Shanghai, China; ^3^ Department of Pharmacy, Fudan University Shanghai Cancer Center Xiamen Hospital, Xiamen, China

**Keywords:** HLX02, trastuzumab, biosimilar, metastatic breast cancer, real-world study

## Abstract

**Background:**

HLX02 is the first China-manufactured trastuzumab biosimilar. Few data are currently available about HLX02 in clinical practice. This study was designed to evaluate the real-world safety and efficacy of HLX02 in patients with HER2-positive metastatic breast cancer (MBC), as well as assessed the effectiveness of switching from trastuzumab originator (Herceptin^®^) to HLX02 during treatment.

**Methods:**

Between April 2021 and October 2022, all patients with HER-2-positive MBC who received at least one cycle of HLX02 at Fudan University Shanghai Cancer Center were included in a retrospective analysis. Patients were divided into two groups: the naïve group (patients treated with HLX02 from the beginning) and the switched group (patients who switched from Herceptin^®^ to HLX02). Efficacy evaluation and adverse events were compared between the two groups.

**Results:**

A total of 124 eligible patients were finally included, with 80 patients (64.5%) in the naïve group, 44 patients (35.5%) in the switched group. The follow-up ranged from 0.7 to 40.2 months, the effectiveness rates were 57.5% in the naïve group and 54.5% in the switched group, respectively (P=0.751). The estimated median progression-free survival (PFS) were 13.70 (95% CI: 8.634–18.766) months and 14.70 (95% CI: 6.684–22.716) months in the naive and switched groups, respectively (P=0.192). Multivariate cox regression analysis suggested that brain metastasis and the current number of treatment lines were independent predictors of MBC PFS. Compared with first-line treatment, second-line treatment and third- or later-line treatment increased the disease risk by 2.095 times (95% CI: 1.043-4.210, P=0.038) and 3.035 times (95% CI:1.751-5.262, P<0.001), respectively. The incidence and distribution of treatment-emergent adverse events (TEAEs) occurrence between the two groups were relatively similar, with no significant statistical difference.

**Conclusions:**

HLX02 demonstrated favorable efficacy and safety in real-world practice comparable to those observed in previous HLX02 studies. Switching between trastuzumab originator and biosimilar for MBC treatment had no impact on efficacy and did not increase safety risks.

## 1 Introduction

Breast cancer remains one of the most common malignancies worldwide. In 2022, 2.3 million women were diagnosed with breast cancer, and 670,000 women died from the disease (https://www.who.int/news-room/fact-sheets/detail/breast-cancer).

Human epidermal growth factor receptor (HER2), a growth factor receptor gene, women with breast cancers that overexpress HER2 have an aggressive form of the disease, with significantly shortened disease-free survival and overall survival (1). Trastuzumab (Herceptin^®^, Genentech/Roche, Inc.), a humanized monoclonal antibody directed against the extracellular domain of HER2, specifically acts on HER2 on cancer cell surfaces and has significantly improved patient prognosis, was approved for treatment of HER2-positive breast cancer and for the treatment of HER2-positive metastatic gastric or gastroesophageal junction adenocarcinoma (2). However, its high cost makes it unaffordable for patients in developing countries such as China (3, 4).

The main advantages of biosimilars were cost savings and lower prices, it is essential to provide oncologists with comprehensive data on the safety and effect, and real-world evidence of biosimilars (5). Biosimilars are developed by different manufacturers, replicating the complex structures and maintaining similar therapeutic efficacy and safety profiles as the original innovator drugs is important (6). At present, many countries are committed to the development of trastuzumab biosimilars, comparing the biosimilars with trastuzumab originator (Herceptin^®^) (7–12). HLX02 (Zercepac^®^, Henlius, Inc.), launched in China in 2020, was the first China-manufactured trastuzumab biosimilar (13–16). It is more cost-effective than Herceptin^®^ in China based on willingness-to-pay thresholds (17). A multicenter real-world has shown HLX02 and Herceptin^®^ to have equivalent efficacy and adverse events in HER-2-positive breast cancer (18). However, real-world data on the safety and efficacy of HLX02 still remain limited, especially regarding switching from Herceptin^®^ to HLX02.

This study aimed to evaluate HLX02’s efficacy and safety based in HER2-positive metastatic breast cancer (MBC), and assess the effectiveness of switching from Herceptin^®^ to HLX02 during treatment. It seeks to provide evidence for the clinical substitution of biosimilars in China.

## 2 Materials and methods

### 2.1 Study design and patients

This study was a retrospective, single-center, non-intervention real-world study. Patients who started therapy naïve to HLX02 and who switched from Herceptin^®^ were collected at Fudan University Shanghai Cancer Center between April 2021 to October 2022. The major inclusion criteria were (a) patients with a pathological diagnosis of HER-2-positive metastatic breast cancer (MBC), HER2 positivity was defined as immunohistochemistry (ICH) 3+ or 2+/fluorescence *in situ* hybridization (FISH) amplification;(b) patients older than 18 years; (c) Eastern Cooperative Oncology Group (ECOG) performance score 0–2. The main exclusion criteria were (a) patients with incomplete medical records, with missing values exceeding 30%; (b) patients with prior or concurrent malignancies (other than thyroid cancer or cancer *in situ* of other organs).

This study was approved by the Ethics Committee of Fudan University Shanghai Cancer Center (No.2021-121-2424).

### 2.2 Data collection

Data variables collected from the patient’s medical records included the following categories: (a) demographic and clinical characteristics, such as sex, age, menopausal status, estrogen receptor status, Ki67 level, metastasis, comorbidities and ECOG performance score; (b) drugs and outcomes, such as trastuzumab utilization patterns, efficacy evaluation; (c) abnormal clinical or laboratory findings, such as nausea, diarrhea, leukopenia, lymphopenia, thrombocytopenia etc. Data and follow-up records were updated as of September 30, 2024.

### 2.3 Treatment and dosage information

In salvage treatment of MBC, the initial loading dose of HLX02 or Herceptin^®^ was 8mg/kg, and the maintenance dose is 6mg/kg once a time, and it is administered once every 3 weeks. The salvage treatment regimen of HLX02 combined with other anti-tumor drugs was determined by the clinical doctor. The study did not intervene.

### 2.4 Assessments and definition of outcomes

Efficacy endpoints were assessed based on imaging reports following Response Evaluation Criteria in Solid Tumors (RECIST 1.1 version). The outcomes were effectiveness rate and the progression-free survival (PFS). In HLX02-naïve patients, if the best overall response of complete response (CR) or partial response (PR) was achieved, HLX02 was considered “effective”. In patients who switched from Herceptin^®^ to HLX02, if the best overall response remains the same as before the switch or improved somewhat, HLX02 was considered “effective” (19). PFS was defined as the time from initiation of HLX02 or Herceptin^®^ treatment until disease progression, including any recurrence or death from any cause.

Safety endpoints were assessed and grated based on National Cancer Institute Common Terminology Criteria for Adverse Events v5.0 grading. The evaluation of adverse events included general adverse events and adverse event of special interest. The predefined adverse event of special interest was cardiotoxicity (such as palpitation, ventricular arrhythmia and reduced left ventricular function) and infusion-related reaction.

### 2.5 Statistical analysis

Normally distributed continuous variables were expressed as mean ± standard deviation, and were calculated by an independent samples t test. Non-normally distributed variables were summarized as median values, range, and were compared by Mann–Whitney U test. Differences between categorical variables were assessed using the chi-square test or Fisher’s exact test. The Kaplan-Meier method was used for PFS analysis, and the log-rank test was used to determine statistically significant variables. Univariate and multivariate analyses were performed with the Cox proportional hazards regression model. Hazard ratios (HR) and 95% confidence interval (95% CI) were determined. A two-sided P-value<0.05 was considered statistically significant. Survival analysis was performed using the Kaplan-Meier method, and comparisons between groups were conducted with the log-rank test. A p-value of less than 0.05 was considered statistically significant. Statistical plotting was performed using GraphPad Prism software (version 10.1.2, GraphPad Software, San Diego, CA, USA). Statistical analyses were performed using SPSS software (version 25.0, SPSS Inc., Chicago, IL, USA).

## 3 Result

### 3.1 Patient characteristics

**Figure 1:**
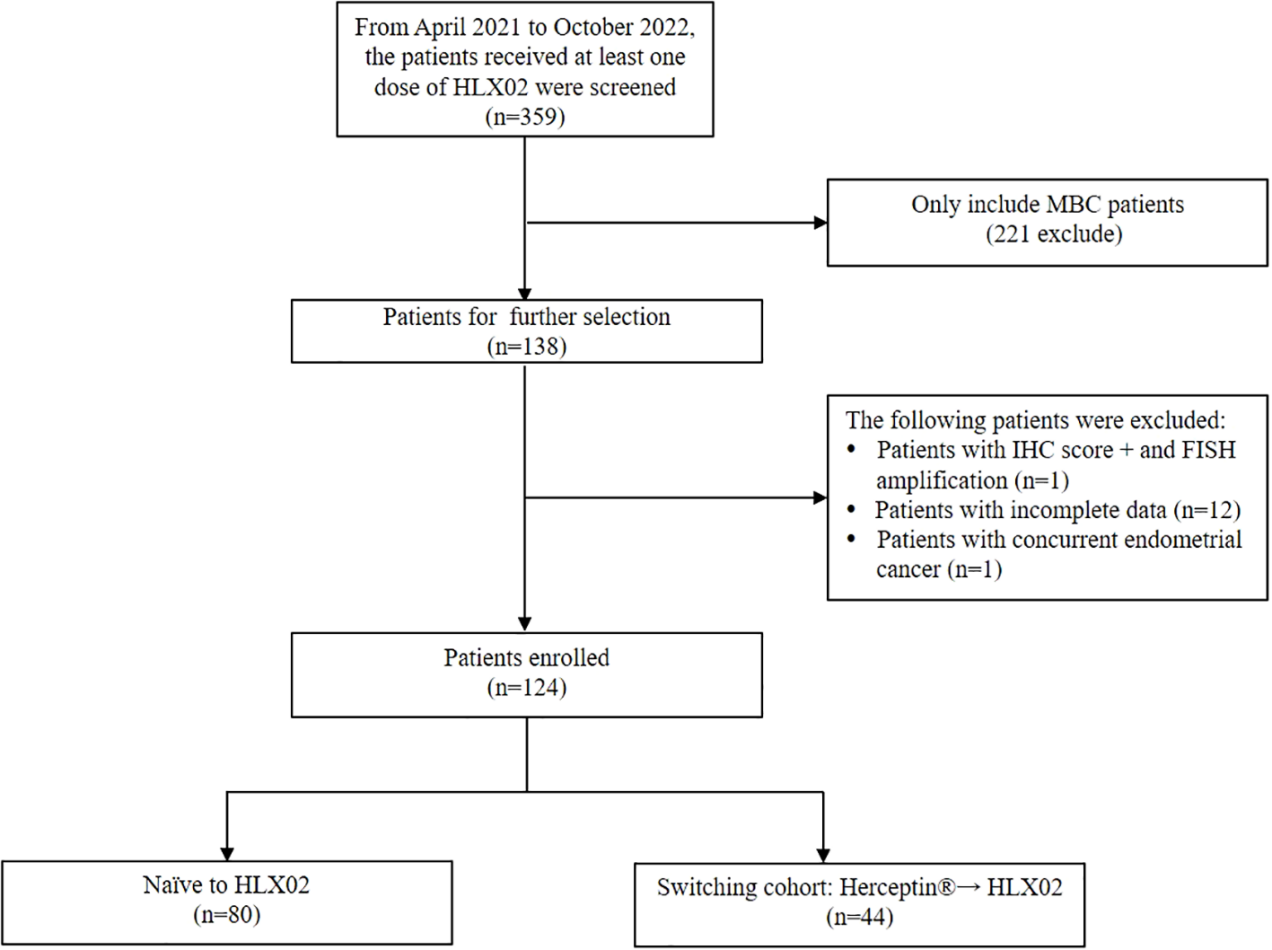
Flow-chart of patient inclusion.

From April 2021 to October 2022, 359 patients received at least one dose of HLX02 were screened (**Figure 1**). Among them, 124 MBC patients were included in our study. Patients were classified into two groups according to their prior trastuzumab treatment status: 80 patients (64.5%) were naïve to HLX02, 44 patients (35.5%) switched from the originator Herceptin^®^ to HLX02, respectively. In the switched group, the median exposure time of Herceptin^®^ and HLX02 was 5.4 months (range, 0.8-51.3) and 5.1 months (range, 0.8-35), respectively. The median cycles of Herceptin^®^ and HLX02 were 7 and 7, respectively.

The demographics and clinical characteristics of the population are presented in **Tables 1** and **2**. All patients were female, the median age was 53 (range, 27-79) years. 78 (62.9%) patients were postmenopausal, 58 (46.8%) patients were ER positive, 46 (37.1%) patients were PR positive. Among them, 77 patients (62.1%) had 1 or 2 sites metastases, 47 patients (37.9%) had 3 or more sites metastases. 76 patients (61.3%), 16 patients (12.9%), 32 patients (25.8%) had who had previously received first-line, second-line, and third- or later-line treatments, respectively. The trastuzumab, pertuzumab and taxanes (THP) was the most commonly used dual-target therapy regimen. There were no significant statistical differences in demographics and clinical characteristic between the naïve group and switched group.

**Table 1:** Demographic and clinical characteristics of patients.

Characteristics	Total (n=124)	Naïve group (n=80)	Switched group (n=44)	P
Age, n (%)				0.191
<53y	59 (47.6)	35 (43.8)	24 (54.5)	
≥53y	65 (52.4)	45 (56.2)	20 (45.5)	
Menopausal status, n (%)				0.298
Premenopausal	46 (37.1)	27 (33.8)	19 (43.2)	
Postmenopausal	78 (62.9)	53 (66.2)	25 (56.8)	
ER status, n (%)				0.239
Positive	58 (46.8)	41 (50.6)	17 (39.5)	
Negative	66 (53.2)	40 (49.4)	26 (60.5)	
PR status, n (%)				0.249
Positive	46 (37.1)	33 (40.7)	13 (30.2)	
Negative	78 (62.9)	48 (59.3)	30 (69.8)	
Ki-67				0.745
≦14%	11 (8.9)	8 (10.0)	3 (6.8)	
>14%	113 (91.1)	72 (90.0)	41 (93.2)	
HER-2 status, n (%)				0.087
IHC 3+	90 (72.6)	54 (67.5)	36 (81.8)	
IHC 2+ and FISH amplification	34 (27.4)	26 (32.5)	8 (18.2)	
Site of metastatic disease, n (%)						
Brain	25 (20.2)	14 (17.5)	11 (25.0)	0.319
Lung	55 (44.4)	39 (48.8)	16 (36.4)	0.184
Bone	63 (50.8)	38 (47.5)	25 (56.8)	0.321
Liver	40 (32.3)	30 (37.5)	10 (22.7)	0.092
Distant lymph node	71 (57.3)	42 (52.5)	29 (65.9)	0.149
Number of metastases, n (%)				0.516
1-2	77 (62.1)	48 (60.0)	29 (65.9)	
≥3	47 (37.9)	32 (40.0)	15 (34.1)	
Comorbidity, n (%)				0.632
No	84 (67.7)	53 (66.2)	31 (70.5)	
Yes	40 (32.3)	27 (33.8)	13 (29.5)	
Baseline electrocardiogram, n (%)				0.405
Normal	86 (69.4)	54 (73.0)	32 (80.0)	
Abnormal	28 (22.6)	20 (27.0)	8 (20.0)	
Missing	10 (8.1)	/	/	
ECOG, n (%)				0.137
0	17 (13.7)	9 (11.2)	8 (18.2)	
1	97 (78.2)	62 (77.5)	35 (79.5)	
2	10 (8.1)	9 (11.2)	1 (2.3)	
LVEF, %	66.5 ± 3.67	67.1 ± 3.66	65.9 ± 3.62	0.142

PR, progesterone receptor; ER, estrogen receptor; ECOG, eastern cooperative oncology group; LVEF, left ventricular ejection fraction.

**Table 2:** Treatment characteristics of patients.

Characteristics	Total (n=124)	Naïve group (n=80)	Switched group (n=44)	P
Current number of treatment lines, n (%)				0.600
1	76 (61.3)	47 (58.8)	29 (65.9)	
2	16 (12.9)	10 (12.5)	6 (13.6)	
≥3	32 (25.8)	23 (28.8)	9 (20.5)	
Target therapy, n (%)				0.688
Single-target	18 (14.5)	11 (13.8)	7 (15.9)	
Trastuzumab + Pertuzumab	93 (75.0)	59 (73.8)	34 (77.3)	
Trastuzumab + Pyrotinib	13 (10.5)	10 (12.5)	3 (6.8)	
Combined chemotherapy regimens, n (%)				0.254
THP	50 (40.3)	27 (33.8)	23 (52.3)	
TCbHP	22 (17.7)	17 (21.2)	5 (11.4)	
HP+others	20 (16.1)	14 (17.5)	6 (13.6)	
H+Pyrotinib+Others	14 (11.3)	11 (13.8)	3 (6.8)	
H+Others	18 (14.5)	11 (13.8)	7 (15.9)	

T, taxanes, including albumin-bound paclitaxel and paclitaxel; H, trastuzumab; P, pertuzumab; Cb, carboplatin.

### 3.2 Efficacy results

**Figure 2:**
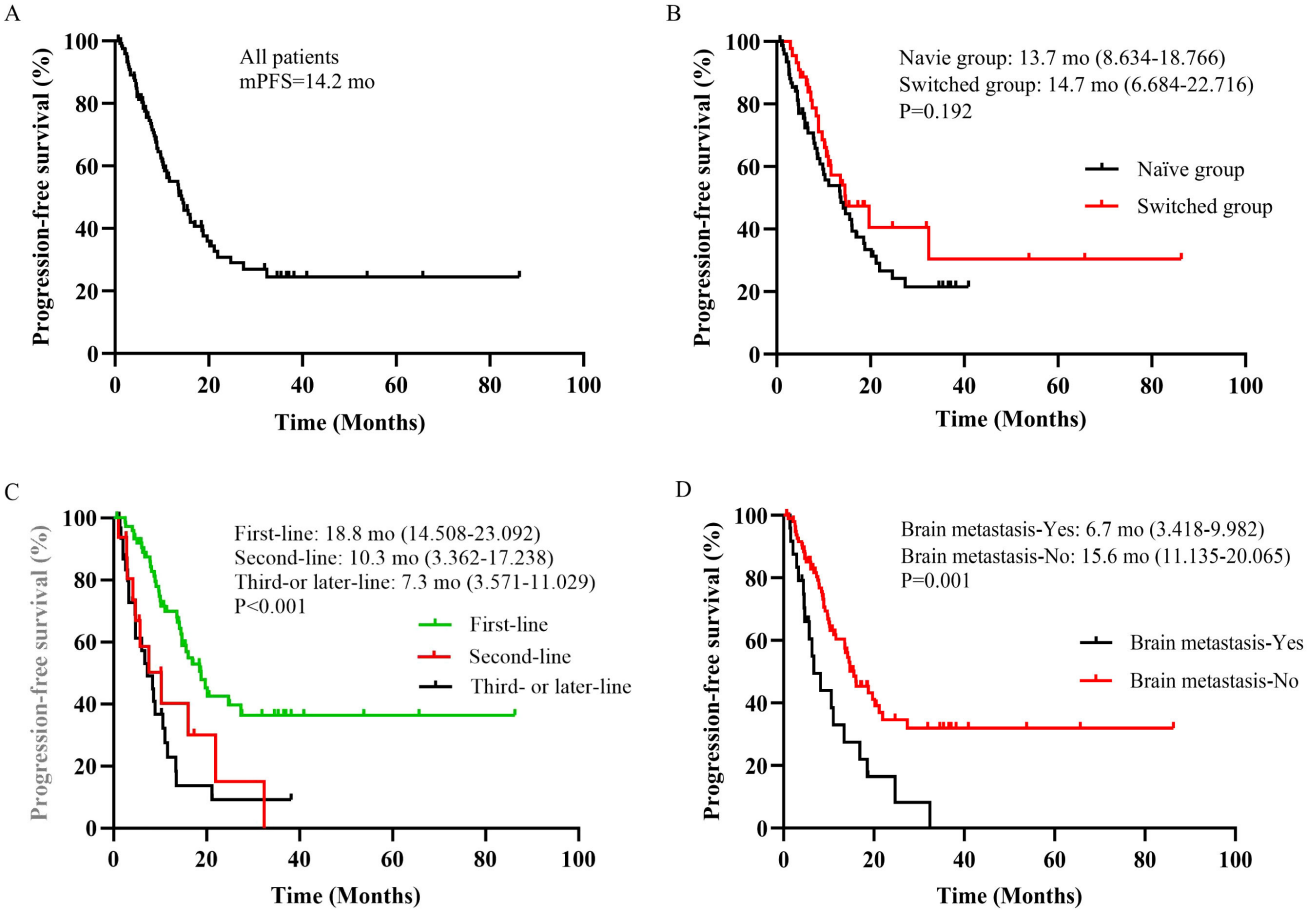
**(A)** Overall cohort. **(B)** Patients stratified by trastuzumab treatment status. **(C)** Patients stratified by current number of treatment lines. **(D)** Patients stratified by brain metastasis. mo, months.

The follow-up ranged from 0.7 to 40.2 months, based on the RECISTv1.1 criteria for clinical efficacy evaluation, no patients achieved CR, 73 patients (58.9%) achieved the best response of PR, 44 patients (35.5%) achieved SD. Among them, 7 patients (5.6%) had PD and one patient eventually died due to disease progression. HLX02 was rated as “effective” in 46 (57.5%) of naïve patients and in 24 (54.5%) of switched patients (P=0.751) (**Table 3**). The median PFS is shown in **Figure 2**, which was 14.2 months (95% CI: 10.5 - 17.9). The results of univariate analysis indicated that the number of metastases, brain metastasis, the number of current treatment lines (second-line vs. first-line, third- or later-line vs. first-line), and treatment regimens (TCbHP vs. THP) were the influencing factors for the survival period of MBC. However, trastuzumab switching during treatment had no impact on the survival period, as shown in **Table 4**. Multivariate Cox analysis suggested that brain metastasis and the number of current treatment lines were the independent predictors of MBC PFS. Compared with first-line treatment, second-line treatment and third- or later-line above treatment increased the disease risk by 2.095 times (95% CI: 1.043-4.210, P = 0.038) and 3.035 times (95% CI: 1.751-5.262, P < 0.001), respectively.

**Table 3:** Efficacy outcomes of two groups.

OutcomeParameter	Total (n=124)	Naïve group (n=80)	Switched group (n=44)
Best overall response, n (%)			
PR	73 (58.9)	46 (57.5)	27 (61.4)
SD	44 (35.5)	28 (35.0)	16 (36.4)
PD	7 (5.6)	6 (7.5)	1 (2.3)
Effectiveness Rate	70 (56.5)	46 (57.5)	24 (54.5)*
95% exact CI	50.1%-67.7%	46.4%-68.6%	46.4%-76.3%
Estimated median PFS (95% CI)	14.2 (10.5-17.9)	13.7 (8.63-18.77)	14.7 (6.68-22.72)

PR, partial response; SD, stable disease; PD, progressive disease.

*HLX02 was considered to be “ineffective” for 3 patients who reported disease progression after switched to HLX02.

**Table 4:** Univariate and multivariate analysis for PFS in MBC patients.

Variables	HR	95%CI	P
Univariate analysis			
Age (≥53y vs. <53y)	1.338	0.831-2.155	0.231
Menopausal status (Postmenopausal vs. Premenopausal)	1.137	0.692-1.868	0.612
ER (Negative vs. Positive)	0.949	0.589-1.528	0.829
PR (Negative vs. Positive)	1.056	0.643-1.734	0.830
Ki-67 (≦14% vs. >14%)	0.861	0.372-1.990	0.726
HER-2 status (IHC 3+ vs. IHC 2+ and FISH amplification)	1.346	0.811-2.234	0.251
Brain metastasis (Yes vs. No)	2.354	1.383-4.008	**0.002**
Lung metastasis (Yes vs. No)	1.198	0.746-1.926	0.455
Bone metastasis (Yes vs. No)	1.236	0.770-1.984	0.381
Liver metastasis (Yes vs. No)	1.154	0.695-1.916	0.580
Metastatic site number (≥3 vs. 1~2)	2.372	1.470-3.829	**<0.001**
Comorbidity (Yes vs. No)	1.138	0.689-1.880	0.613
ECOG score			
(1 vs 0)	2.074	0.941-4.574	0.071
(2 vs 0)	2.593	0.870-7.729	0.087
Number of current treatment lines			
(Second-line vs. First-line)	2.431	1.232-4.798	**0.010**
(Third-or later-line vs. First-line)	3.345	1.955-5.723	**<0.001**
Treatment regimen			
(TCbHP vs. THP)	2.072	1.086-3.952	**0.027**
(HP+Others vs THP)	1.274	0.593-2.736	0.534
(H+Others vs THP)	1.437	0.701-2.944	0.322
(H+Pyrotinib vs THP)	1.362	0.629-2.950	0.433
Trastuzumab treatment status			
(Naïve group vs. Switched group)	0.715	0.430-1.188	0.195
Multivariate analysis			
Brain metastasis (Yes vs. No)	1.827	1.052-3.172	**0.032**
Number of current treatment lines			
(Second-line vs. First-line)	2.095	1.043-4.210	**0.038**
(Third- or later-line vs. First-line)	3.035	1.751-5.262	**<0.001**

PR, progesterone receptor; ER, estrogen receptor; PFS, progression-free survival; HR, hazard ratio; CI, confidence interval; T, taxanes, including albumin-bound paclitaxel and paclitaxel; H, trastuzumab; P, pertuzumab; Cb, carboplatin. Bold values indicate P-value < 0.05, representing statistically significant differences.

### 3.3 Safety

During the study period, a total of 375 treatment-emergent adverse events (TEAEs) were occurred, involving 112 patients (90.3%). The severity of most TEAEs was grade 1-2, and 36 patients (29.0%) occurred 120 episodes of grade 3–4 TEAEs. As shown in **Table 5**, the incidence of any-grade TEAEs between the naïve group and the switched group were similar, and there were no significant differences (72 patients [90.0%] vs. 40 patients [90.9%], P=0.870). The incidence of grade 3–4 TEAEs was higher in the naïve group than that in the switched group, but the difference was not statistically significant (26 patients [32.5%] vs. 10 patients [22.7%], P=0.251). The most common (≥10%) TEAEs were hematological toxicity and liver function abnormalities, exhibiting anemia (51.2% vs 65.9%, P=0.115), increased ALT (37.5% vs 36.4%, P=0.900), leukopenia (31.2% vs 18.2%, P=0.115), increased AST (28.8% vs 29.5%, P=0.926), neutropenia (25.0% vs 20.5%, P=0.660), but there was no significant difference between the two groups. One death case was occurred due to disease progression, but it was recorded as not related to HLX02. No new safety signals detected during the real-world practice.

**Table 5:** Summary of adverse events.

Adverse Events	Navie group (n=80)	Switched group (n=44)	P
TEAEs, n(%)			
Any grade	72 (90.0)	40 (90.9)	0.870
Grade 3-4	26 (32.5)	10 (22.7)	0.251
AEs occurred in ≥10% patients in either group, n(%)			
Anemia	41 (51.2)	29 (65.9)	0.115
Increased ALT	30 (37.5)	16 (36.4)	0.900
Leukopenia	25 (31.2)	8 (18.2)	0.115
Increased AST	23 (28.8)	13 (29.5)	0.926
Neutropenia	20 (25.0)	9 (20.5)	0.660
Increased alkaline phosphatase	13 (16.2)	11 (25.0)	0.238
Thrombocytopenia	9 (11.2)	3 (6.8)	0.536
Hyperuricemia	8 (10.0)	10 (22.7)	0.054
Fatigue	7 (8.8)	3 (6.8)	1.000
Diarrhea	8 (10.0)	2 (4.5)	0.492
Decreased appetite	5 (6.2)	2 (4.5)	1.000
Hyperbilirubinemia	5 (6.2)	5 (11.4)	0.324
AEs of special interest occurring in ≥ 5% of patients, n(%)			
Infusion-related reaction	7(8.8)	5(11.4)	0.753
Cardiotoxic effects	2(2.5)	3(6.8)	0.346

TEAE treatment-emergent adverse event; ALT, alanine aminotransferase, AST, aspartate aminotransferase.

It is worth noting that infusion-related reaction and cardiotoxicity were reported, a total of 12 patients developed infusion-related reactions, with 7 patients (8.8%) in the naïve group, and 5 patients (11.4%) in the switched group (P=0.753). A total of 5 patients reported cardiotoxicity, with 2 patients (2.5%) in the naïve group and 3 patients (6.8%) in the switched group, and there was no significant difference between the two groups (P=0.346).

## 4 Discussion

This study evaluated the efficacy and safety of HLX02 in patients with MBC based on real-world clinical data, and provided evidence for the effectiveness of trastuzumab switching during treatment.

As the first China-manufactured trastuzumab biosimilar, HLX02 is approved in Europe (EU) and China, Zhou et al. confirmed that HLX02 is bioequivalent to the originator Herceptin^®^, with similar safety and immunogenicity profiles (16). Xu et al. shows that the objective response rate (ORR) at week 24 (71.3%), PFS (11.7 months), and OS (not reached) observed in the HLX02 treatment group (18). In addition, after a median follow-up duration of 35.0 months, 39.5% patients had died in the HLX02 group, median overall survival (OS) was 37.3 months, with a 3-year OS rate of 57.5%. Median PFS at this long-term follow-up assessment was 11.7 (95% CI 11.5, 12.1) months for the HLX02 group (15).

However, there are few studies on the real-world clinical application of HLX02 in the treatment of HER-2 positive MBC, especially in combination with other antitumor agents, such as pertuzumab. *Deng* et al. demonstrated that 32 patients (86.5%) achieved CR, 2 patients (5.4%) achieved PR in the HLX02 group (20). However, as the majority (6/96, 93.8%) of the included patients were in the early stage, this study has certain limitations for MBC. Our study focused on patients with MBC, and the results showed that 73 patients (58.9%) achieved PR, but no patients achieved CR. After a median follow-up of 0.7-40.2 month, the median PFS for HLX02 first-line treatment was 18.8 months, and the median PFS for second-line treatment was 10.3 months, the median PFS of third- or later-line treatment was 7.3 months. In previous phase III studies, the median PFS of trastuzumab or trastuzumab biosimilar combined with taxanes in first-line treatment of MBC was 10.6-12.8 months (8, 12, 18, 21, 22). At present, there are few efficacy data about second-line or later treatment of HLX02 in MBC. In the Phase II clinical trial of HLX02, 45 patients with HER-2 positive MBC were enrolled to receive HLX02, pertuzumab and physician selection chemotherapy, 12 patients (26.7%) were treated with second-line therapy, 33 patients (73.3%) were treated with third- or later-line treatment (23). Median follow-up was 1.2-43.9 months, the median PFS for second-line treatment was 6.26 months (range: 0-18.9), and the median PFS for third- or later-line treatment was 7.6 months (range: 4.8-10.3) (23). The results of our study were slightly different from the Phase II/III trial. The possible reasons are as follows: (1) the efficacy results might be affected by the characteristics of the enrolled patients, previous treatment experiences, and methodological factors (such as dosing regimens and efficacy assessment); (2) in the treatment regimen of the phase III trial, pertuzumab was not added. However, in real-world practice, more than 75% of patients received “trastuzumab plus pertuzumab” dual-targeted therapy, which to some extent increased the PFS.

In this study, 44 patients (35.5%) MBC patients experienced switching between trastuzumab originator and biosimilar. At present, the available research data are limited regarding whether the switching between the originator and biosimilar would have an impact on efficacy and safety. The LILAC study reported that among 342 HER-2 positive EBC patients who received neoadjuvant Herceptin^®^ treatment, 171 patients switched to trastuzumab biosimilar ABP 980 during the postoperative adjuvant treatment (24). In terms of prognosis, there was no significant statistical difference in disease progression, recurrence or mortality between the switched group and the non-switched group (HR = 0.48, 95% CI: 0.181 - 1.292); in terms of safety, there was no significant statistical difference in the overall AE incidence (26.3% vs. 22.8%, P > 0.05) and the incidence of severe AEs (7.6% vs. 6.4%, P > 0.05) between the two groups after a follow-up of 12.0 months; in terms of immunogenicity, the positive rate of anti-drug antibodies in the switched group was 1.2%, which was higher than 0.6% in the non-switched group (24). Overall, after the one-way switch (originator→biosimilar), the efficacy, safety and immunogenicity indicators of HER-2 positive breast cancer patients did not undergo significant changes. We also conducted a preliminary exploration on the impact of trastuzumab switching on outcome indicators during the research. No significant differences were observed in effectiveness rates for patients in the naïve group or in the switched group. Furthermore, the univariate analysis showed that trastuzumab switching had no impact on PFS (P=0.195).

One strength of our study is that it included the heterogeneous characteristics of clinical practice, that is more representative of entire patient population than the carefully selected individuals in clinical trials. A network meta-analysis evaluated efficacy and serious adverse reactions among various trastuzumab biosimilars and trastuzumab originator. The cumulative ranking curve (SUCRA) probability indicated that the ORR from best to worst was CT-P6, Herceptin, HLX02, PF-05280014, R-TPR-016, BCD-022, MYL-1401O, SB3. There was no statistical difference in both ORR and pathological complete response (pCR) of various trastuzumab biosimilars and Herceptin except SB3 (25). According to the result, HLX02 performs might be an optional trastuzumab biosimilar compared with others in China.

The SUCRA probability indicated that severe AEs from best to worst was MYL-1401O, Herceptin, PF-05280014, SB3, HLX02, BCD-22, CT-P6 (25). Our study showed that the safety profiles were comparable with the known safety profiles of trastuzumab in patients with breast cancer (18, 20). Anemia, increased ALT and AST, leukopenia, neutropenia, increased alkaline phosphatase and thrombocytopenia were the most common TEAEs identified in this study. These events were also consistent with previous studies of trastuzumab biosimilars (8, 12, 19, 20). There were no notable differences between the naïve group and the switched group regarding the type, incidence, or severity of TEAEs. Trastuzumab has been reported in most research as related to increased risks of cardiac toxicity (26, 27). Thus, cardiotoxicity in the two groups were carefully assessed. The frequency of related events was low and similar between the two groups (2 vs. 3 patients), and without significant differences in this study.

The irrational use can be found both in resource-abundant regions and in resource-limited regions in China. A study showed the patients who lived in areas with a relatively high gross domestic product were more likely to receive trastuzumab originator than those in areas with a lower gross domestic product (28). In developing countries such as China, where biopharmaceuticals often limit patient access due to high costs, biosimilars provide an additional treatment option for enabling patient access, the introduction of biosimilars into clinical practice is necessary to sustainably reduce the healthcare burden. Treatment with biosimilars is not only a direct cost-saving approach, but also drives the clinical practice of new therapies and drugs (29). This study offers reliable real-world evidence for assessing the quality and safety of HLX02 as a crucial foundation for future evaluations. Switching to different trastuzumab combinations regimens for cancer treatment had no effects on PFS and did not increase safety risks. These real-world findings could help to optimize HER-2 therapy in advanced breast cancer, especially in regions with limited access to these expensive targeted drugs.

This study has several limitations. Firstly, as it utilizes retrospective real-world data with limited sample of patients using HLX02. Limited sample may lead to low statistical power of the association analysis, so it is necessary to expand the sample size and conduct a large-scale clinical trial with multi-center cooperation. Future studies with larger sample sizes also could validate stratified analysis based on the co-administered drugs, such as adjunctive medications, target therapy or combined with different chemotherapy. Secondly, patients are recruited from single centers and only included Chinese populations. As such, the findings are probably representative in this region, may not be generalizable globally. Finally, the patients received trastuzumab in combination with other drugs during the treatment, it may interfere whether some adverse events were caused by trastuzumab.

## 5 Conclusion

This study provided the real-world use of trastuzumab originator and its biosimilars (HLX02), the safety and efficacy of biosimilars were confirmed. These findings offered valuable information for implementation of switching from trastuzumab originator to a biosimilar.

## Data Availability

The original contributions presented in the study are included in the article/supplementary material. Further inquiries can be directed to the corresponding authors.
